# Serum Immune Responses to Group A Streptococcal Antigens following Pharyngeal Acquisitions among Children in Cape Town, South Africa

**DOI:** 10.1128/msphere.00113-23

**Published:** 2023-05-08

**Authors:** M. Taariq Salie, Babu Muhamed, Kélin Engel, Kimona Rampersadh, Rezeen Daniels, Lwazi Mhlanti, Thomas A. Penfound, Craig A. Sable, Liesl J. Zühlke, James B. Dale, Mark E. Engel

**Affiliations:** a Department of Medicine (AFROStrep Research Initiative) and Cape Heart Institute, Faculty of Health Sciences, University of Cape Town, Cape Town, South Africa; b Department of Medicine, University of Tennessee Health Science Center, Memphis, Tennessee, USA; c Children’s National Health System, Washington, DC, USA; d South African Medical Research Council, Cape Town, South Africa; e Division of Paediatric Cardiology, Department of Paediatrics, Faculty of Health Sciences, University of Cape Town, Cape Town, South Africa; The University of Texas Medical Branch at Galveston

**Keywords:** ELISA, GAS shared antigens, longitudinal, M protein, South Africa, group A *Streptococcus* (GAS), immune response, vaccines

## Abstract

There is limited information on the human immune response following infection with group A *Streptococcus* (Strep A). Animal studies have shown, in addition to the M protein, that shared Strep A antigens elicit protective immunity. This study aimed to investigate the kinetics of antibody responses against a panel of Strep A antigens in a cohort of school-aged children in Cape Town, South Africa. Participants provided serial throat cultures and serum samples at two-monthly follow-up visits. Strep A recovered were *emm*-typed, and serum samples were analyzed by enzyme-linked immunosorbent assay (ELISA) to assess immune responses to thirty-five Strep A antigens (10-shared and 25-M peptides). Serologic evaluations were performed on serial serum samples from 42 selected participants (from 256 enrolled) based on the number of follow-up visits, the frequency of visits, and throat culture results. Among these, there were 44 Strep A acquisitions, 36 of which were successfully *emm*-typed. Participants were grouped into three clinical event groups based on culture results and immune responses. A preceding infection was most convincingly represented by a Strep A-positive culture with an immune response to at least one shared antigen and M peptide (11 events) or a Strep A-negative culture with antibody responses to shared antigens and M peptides (9 events). More than a third of participants demonstrated no immune response despite a positive culture. This study provided important information regarding the complexity and variability of human immune responses following pharyngeal acquisition of Strep A, as well as demonstrating the immunogenicity of Strep A antigens currently under consideration as potential vaccine candidates.

**IMPORTANCE** There is currently limited information regarding the human immune response to group A streptococcal throat infection. An understanding of the kinetics and specificity of antibody responses against a panel of Group A *Streptococcus* (GAS) antigens will serve to refine diagnostic approaches and contribute to vaccine efforts, which together will serve to reduce the burden of rheumatic heart disease, a major source of morbidity and mortality especially in the developing world. This study, utilizing an antibody-specific assay, uncovered three patterns of response profiles following GAS infection, among 256 children presenting with sore throat to local clinics. Overall, the response profiles were complex and variable. Of note, a preceding infection was most convincingly represented by a GAS-positive culture with an immune response to at least one shared antigen and M peptide. Also, more than a third of participants demonstrated no immune response despite a positive culture. All antigens tested were immunogenic, providing guidance for future vaccine development.

## INTRODUCTION

Group A Streptococcus (Strep A) is a strict human pathogen that causes a variety of infections and diseases such as pharyngitis, scarlet fever, and impetigo, as well as more severe diseases such as necrotizing fasciitis, streptococcal toxic shock syndrome (STSS), and the poststreptococcal autoimmune sequelae acute rheumatic fever (ARF), rheumatic heart disease (RHD), and poststreptococcal glomerulonephritis (PSGN) (1). Despite being one of the most studied bacterial pathogens, there is not currently a licensed Strep A vaccine. One impediment to vaccine development is that there is insufficient information regarding human immune responses to Strep A antigens following natural infection. Additionally, there is not an established immune correlate of protection.

Most previous studies have focused on immune responses to the M protein because of its central role as a determinant of virulence and because M antibodies are associated with protection against infection in animals (2, 3) and humans (4). The multiplicity of genomically distinct M types (now >200) has prompted the development of multivalent M protein-based subunit vaccines to achieve sufficient coverage of the most common *emm* types (5, 6). Alternative approaches to the development of vaccines with potentially broad coverage involve the identification of protective antigens that are shared by all or most Strep A (7). While most of the shared antigens being considered vaccine candidates have demonstrated variable levels of protective immunogenicity in animal models, the role of these antigens in human immunity to Strep A infections is unknown.

In a previous report, we demonstrated highly variable antibody responses to shared antigens and more consistent, yet incomplete, responses to M peptides in participants from the United States that were enrolled in a study of pediatric autoimmune neuropsychiatric disorders associated with Strep A infections (PANDAS) (8). Limitations of the previous study included the limited number of infecting *emm* types, the fact that the participants had previously been identified as meeting criteria for a diagnosis of PANDAS, and that they were from geographic regions at very low risk for ARF and RHD.

Our recent systematic review highlighted limitations in using upper limit of normal (ULN) values of immune responses as a baseline for determining a positive reaction to an antigen given the natural variation of titers across population groups (9). Thus, there is value in evaluating whether a change in antibody level by itself could serve in determining positive responses to antigens following a Strep A infection, regardless of antibody concentration within populations.

The current study was designed to determine the kinetics and antigen specificity of antibody responses to 10 shared Strep A antigens and twenty-five M peptides in children with new infections from Cape Town, South Africa, where ARF and RHD are highly prevalent (10 to 12). The longitudinal clinic-based protocol was designed to enroll children who had throat cultures and serum samples obtained at 2-month intervals over a 24-month period. The Strep A isolates were *emm*-typed, and enzyme-linked immunosorbent assays (ELISAs) were performed to assess changes in antibody levels over time. Overall, the results show that the human immune responses following Strep A acquisition were quite variable. Despite the lack of concrete and universal immune response patterns following the acquisition of Strep A, the results highlight the complexities of the bacteria/host interactions. Further, a subset of participants displayed immune responses that may contribute to our understanding of protective immunity. Finally, we present limitations for consideration in future studies.

## RESULTS

Two hundred and fifty-six children were enrolled in the study. The protocol specified a two-monthly interval of follow-up for 24 months. However, the average interval between visits for all participants was 89 days (range, 4 to 617 days). Ninety-one participants withdrew or were lost to follow-up (LTFU). The average number of visits per patient was 3.4. Excluding withdrawals or LTFU after the initial enrollment visit, there was an average of 4.5 visits per patient, with the number of subsequent visits ranging from 2 to 11 for the study period.

Prevalence of β-hemolytic streptococci (βHS) and Strep A among those seeking treatment for pharyngitis at time of enrollment into the study was 70/256 (27.3%) and 54/256 (21.1%), respectively. Overall, 124 βHS isolates were recovered from enrollment and follow-up visits (Strep A = 83, group C *Streptococcus* = 25, group G *Streptococcus* = 16). Sixty-eight of the Strep A cultures were successfully *emm*-typed, while the remaining isolates failed to yield sequence data (*n* = 9), or a pure Strep A culture from the National Health Laboratory Service (NHLS) was never received (*n* = 2) or produced no growth on blood agar plates during culture (*n* = 4). The sixty-eight successfully sequenced Strep A were isolated from 60 participants, of which 46 were obtained upon entry to the study and 22 were new Strep A acquisitions during the study.

### *emm* type prevalence and potential vaccine coverage.

Twenty-seven different *emm* types were obtained throughout the study (Fig. S1, available at https://doi.org/10.25375/uct.21510186.v3). The most prevalent *emm* type was *emm*1 (*n* = 10), followed by *emm*12 (*n* = 7) and *emm*49 (*n* = 6). When grouped according to *emm* clusters, cluster E3 (*n* = 19) was represented most frequently, followed by A-C3 (*n* = 10), E6 and E4 (*n* = 8), and A-C4 and D4 with 7 isolates each. The theoretical coverage of the 30-valent vaccine in this high-risk population was estimated to be 63.2% (*n* = 43) of Strep A isolates, which would increase to 72.1% (*n* = 49) considering previously published *emm* types that were cross-opsonized by 30-valent vaccine antisera (5).

### Strep A acquisitions and symptoms.

Seventy-two participants provided a total of 83 positive Strep A cultures. Of these, 26/83 (31.3%) were obtained postenrollment, and the majority (23/26, 88.5%) were isolated from asymptomatic participants. The most common physical examination findings were tonsillar swelling (*n* = 55), hoarseness (*n* = 14), anterior cervical node >1.5 cm in diameter (*n* = 14), and a temperature above 38°C (*n* = 13). Children were administered antibiotics according to standard of care at the community clinics (penicillin V or amoxicillin, 250 mg/500 mg). Sixty-nine participants received antibiotics throughout the study (Table S1, available at https://doi.org/10.25375/uct.21510186.v3).

### Immune responses to Strep A antigens.

Serologic evaluations were performed on serial serum samples from 42 participants that were selected based on the number of follow-up visits, the frequency of visits, and throat culture results. The average number of follow-up visits within this group was 4.8 (range 2 to 11). Forty participants had one or more positive throat cultures during the study period, and the remaining two participants had negative throat cultures over five visits.

A total of 202 serum samples were assayed for antibody levels against 10 shared Strep A antigens and 25 N-terminal M peptides (Table 1). During the initial review of the serologic results, it was apparent that immune responses were highly variable and no clear patterns emerged. To facilitate analysis of the data, throat culture results and immune responses to shared antigens and M peptides were used to group the data into three clinical events (Table 2). Events in group 1 were defined by a positive throat culture for Strep A and immune responses to at least one shared antigen and/or an M peptide (*n* = 16). Group 2 was defined by a positive Strep A culture but no significant antibody responses to any of the shared antigens or M peptides (*n* = 28). Group 3 events were characterized by a negative culture for Strep A and immune responses to at least one shared antigen and/or an M peptide (*n* = 21). Among the 40 participants in this cohort, 65 clinical events were defined by these criteria (Table 2). Additionally, in group 1, seven participants were symptomatic during infection and of these, three had responses to both M peptide and shared antigens. Twenty-two of 28 participants were symptomatic in group 2, and only two had symptoms in group 3.

**TABLE 1:** Group A streptococcus antigens evaluated^*a*^

Antigen	Bacterium location	Function	Reference(s)
Synthetic N-terminal M peptides (25 M types)	Cell surface	Opsonic epitopes	13
Mrp2, 4, and 49 (N-terminal) and Mrp4B (intact protein)	Cell surface	Opsonic epitopes	14
SLO	Secreted	Hemolysin	15
DNaseB	Secreted	Degrades neutrophil nets	15
SCPA	Cell surface and secreted	Cleaves C5a	33, 34, 35
SpyCEP	Cell surface and secreted	Cleaves IL-8	36, 37
SSE	Secreted	Tissue invasion	38
SpyAD	Cell surface	Cell division and adhesion	39

a DNaseB, DNase B; IL-8, interleukin 8; Mrp, M-related proteins; SCPA, C5a peptidase; SLO, streptolysin O; SpyCEP, serine.

**TABLE 2:** Clinical events according to throat culture results and immune responses^*a*^

Clinical event groups	Culture	no. of events	Immune response	
			Shared ags + M peptide,	Shared ags
1	+	16 (7)	11 (3)	16 (6)
2	+	28 (22)	0	0
3	−	21 (2)	9	21 (2)

a The 2 participants with negative cultures and no immune responses were not included. (Number of symptomatic participants in brackets).

**FIG 1:**
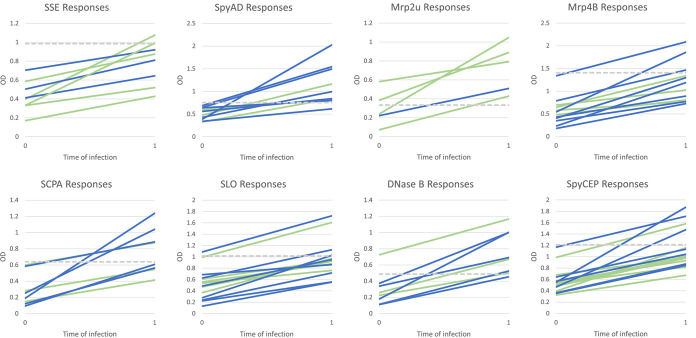
Changes in antibody levels against shared antigens between the index visit and the next visit of participants included in event groups 1 and 3. The mean time between visits was 73.8 days (min = 34 days, max = 130 days) for group 1 and 68.4 days (min = 59 days, max = 84 days) for group 3. Group 1 in green and group 3 in blue, ULN in gray.

Antibody levels (Table S2, available at https://doi.org/10.25375/uct.21510186.v3) in serial serum samples from each participant were considered either new responses, which in combination with throat culture results defined the clinical event group, or as preexisting elevated antibody levels using the criteria described above. To assess the overall immunogenicity of Strep A shared antigens, the total number of events with either new antibody responses or elevated levels of antibodies was evaluated (Table 3). The frequency of antibody responses was highest against Mrp4B, which is the full-length Mrp4 protein containing semiconserved N-terminal epitopes and highly conserved C-terminal sequences. The frequency of antibody responses against SpyCEP, SLO, SSE, SpyAD, and SCPA were observed in decreasing frequency (Table 3). Although M-related proteins are expressed by the majority of Strep A *emm* types, their N-terminal sequences are semiconserved among a limited number of Mrp types (16). As expected, antibodies against Mrp2U and Mrp4U, the N-terminal peptides that define the Mrp type, were observed less frequently, and no serum samples contained antibodies against Mrp49U, which is a less prevalent Mrp type (14). The relative increases in antibody levels against shared Strep A antigens in clinical event groups 1 and 3 are shown in Fig. 1. It is clear that these responses are variable for groups 1 and 3, respectively, where a rise in titer illustrates a positive immune response, and therefore, provides evidence of a true Strep A infection.

**TABLE 3:** New antibody responses and pre-existing high antibody levels against Strep A antigens according to clinical event group (*n* = 65)

	SSE	SpyAD	Mrp4U	Mrp2U	Mrp49U	Mrp4B	SCPA	SLO	DNaseB	SpyCEP	M peptide
New antibody responses (%)											
GRP 1 (*n* = 16)	5 (31.3)	3 (18.8)	0	4 (25)	0	4 (25)	3 (18.8)	6 (37.5)	3 (18.8)	11 (68.8)	11 (68.8)
GRP 2 (*n* = 28)	0	0	0	0	0	0	0	0	0	0	0
GRP 3 (*n* = 21)	3 (14.3)	7 (33.3)	0	1 (4.8)	0	10 (47.6)	5 (23.8)	12 (57.1)	5 (23.8)	9 (42.9)	9 (42.9)
Total	8 (12.3)	10 (15.4)	0	5 (7.7)	0	14 (21.5)	8 (12.3)	18 (27.7)	8 (12.3)	20 (30.8)	
Pre-existing high antibody levels (%)											
GRP 1 (*n* = 16)	1 (6.3)	0	0	1 (6.3)	0	4 (25)	1 (6.3)	1 (6.3)	1 (6.3)	2 (12.5)	5 (31.3)
GRP 2 (*n* = 28)	10 (35.7)	9 (32.1)	0	1 (3.6)	0	11 (39.3)	5 (17.9)	13 (46.4)	5 (17.9)	10 (35.7)	14 (50)
GRP 3 (*n* = 21)	4 (19.1)	3 (14.3)	2 (9.5)	0	0	10 (47.6)	1 (4.8)	3 (14.3)	0	5 (23.8)	6 (28.6)
Total	15 (23.1)	12 (18.5)	2 (3.1)	2 (3.1)	0	25 (38.5)	7 (10.8)	17 (26.2)	6 (9.2)	17 (26.2)	

### Assessment of immune responses within clinical event groups.

New antibody responses to N-terminal M peptides were observed in 20 group 1 and 3 clinical events, which was part of the criteria used to define these groups. Within these groups, there were new antibody responses against 15 different M peptides (Table S2). Three individuals mounted immune responses against more than one M peptide, which may have represented multiple infections during the intervening period between serum samples. Within event group 1, the M peptide antibody response was not always consistent with the *emm* type of the throat culture isolate (Table S2). Thirteen of 16 Strep A isolates were successfully *emm*-typed, and 5 of the M peptide antibody responses matched the *emm* type recovered at the start of the clinical event. Eleven of the 20 events included in clinical event groups 1 and 3 had preexisting high levels of antibody against 14 different M peptides. In total, 18 of 40 participants had preexisting high levels of M peptide antibodies against 14 different *emm* types (Table S2). Four individuals had preexisting antibodies against the same *emm* type that was recovered from the pharynx. Three of these were observed in clinical event group 2, and one was a group 1 event.

**FIG 2:**
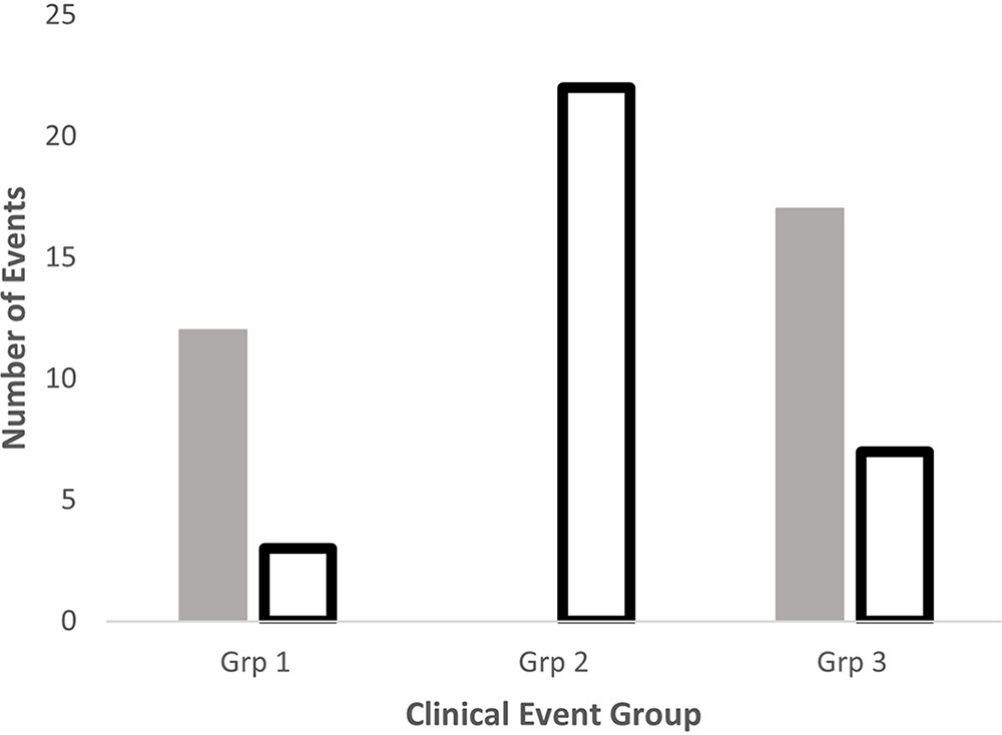
Comparison of clinical events exhibiting preexisting high levels of antibodies (open bars) against shared group A *Streptococcus* (GAS) antigens (SSE, SpyAD, SCPA, SLO, and SpyCEP) versus those without preexisting high levels of antibodies (gray bars). Grp 1 versus Grp 2: *P* < 0.0001; Grps 1 and 3 versus Grp 2: *P* < 0.0001; Grp 3 versus Grp 2: *P* < 0.0001; Grp 1 versus Grp 3: *P* = 0.0021, chi square.

Based on the observations above, clinical event groups 1 and 3 are most consistent with symptomatic or asymptomatic Strep A infections, with predicted and temporally related antibody responses to shared antigens and/or M peptides (Fig. 1, Fig. S2, available at https://doi.org/10.25375/uct.21510186.v3). Event group 2 was defined based on the absence of immune responses to shared antigens or M peptides, despite a positive throat culture for Strep A. None of the event groups was defined using the criterion of preexisting high levels of antibodies against shared antigens. However, tabulating events in each group with and without high levels of antibodies against five shared antigens (SpyCEP, SLO, SpyAD, SCPA, SSE) revealed significant differences between event group 2 and other event groups (Fig. 2). Events in group 2, which were all associated with a positive throat culture but no immune responses, may represent an association between Strep A acquisitions without infection and preexisting elevated levels of antibodies against one or more shared antigens.

## DISCUSSION

This longitudinal study provides support for the utility of Strep A-shared antigens and M peptides in studying human immune responses in children from an area of endemicity in Cape Town following Strep A acquisition. We conclude that (i) there has been a shift in the molecular epidemiology of Strep A in the population compared to a previous study, (ii) individuals demonstrate Strep A-specific antibody responses in the absence of symptomatic infection, (iii) the Strep A antigen-specific serum antibody responses following pharyngeal acquisitions are complex, and (iv) compared to subjects with antibody responses consistent with Strep A infection, the cohort of participants with positive throat cultures but no antigen-specific antibody responses were more likely to have preexisting elevated antibody levels against five shared Strep A antigens.

Our study reports both a temporal and continental distribution in *emm* type epidemiology. In our study conducted a decade previously (17), the more prevalent *emm* types included *emm*48 (*n* = 17); *emm*12 (*n* = 13); *emm*4 (*n* = 11); *emm*89 (*n* = 9); and *emm*94 (*n* = 9). In this study, only *emm*12 ranks among the most prevalent *emm* types isolated. Compared with the distribution of *emm* types within Africa, we affirm clear differences compared with those reported in a recent prevalence systematic review (18) where *emm*18, *emm*65, *emm*75, *emm*76, and *emm*81 are among top *emm* types reported. The changing molecular epidemiology of Strep A infections is consistent with previous studies that showed *emm*-type replacements within large (19) and smaller (20) geographic regions.

### Evidence for asymptomatic immunologically significant infections.

The entire data set comprises 68 successfully sequenced Strep A acquisitions (recovered from 60 participants), of which, 26 were not recovered from the enrollment visit and were defined as new Strep A acquisitions. Of these, only three had symptoms prior to the isolation of Strep A. For event groups 1 and 3, 16/20 experienced new Strep A acquisitions by either culture, immune responses, or both, and none of those participants reported symptoms of sore throat. In the current study, 26/55 Strep A-positive participants (53 = culture, 2 = immune responses) were correctly identified on enrollment as needing treatment by clinical decision rules. However, 19 participants prescribed antibiotics were Strep A-negative. Additionally, in event groups 1 and 3, which most likely represented true infections, only 2/20 participants received antibiotic treatment.

Previous studies have found that a large proportion of Strep A acquisitions were asymptomatic (8, 9, 21), a finding confirmed by the immunological evidence provided in the current study. This observation is particularly relevant in the context of primary prevention of ARF, where asymptomatic immunologically significant Strep A infections could be triggering ARF, given no indication for the prescription of primary antibiotics. Together with our findings, there is support for the notion that asymptomatic Strep A infection may be driving RHD, presenting major challenges in efforts to prevent ARF and RHD through primary prevention with antibiotic treatment of pharyngitis.

All of the shared Strep A antigens used in this study, with the exception of Mrp49U, were immunogenic in at least one participant. These results are consistent with a previous study that identified specific antibodies present in pooled intravenous immunoglobulin therapy (IVIG) against many of the antigens tested in the current study (22). Across all clinical event groups, the frequency of immune responses to the six shared non-Mrp antigens was highest against SpyCEP > SLO > SpyAD > DNaseB > SCPA and SSE. We believe that clinical event groups 1 and 3 demonstrate the most convincing evidence of the immunologically significance of Strep A, even in asymptomatic participants. The immunogenicity of shared antigens in the current study differs somewhat from our previous study (8), which showed that increases in antibody levels following new Strep A acquisitions were observed most frequently against DNaseB and SpyAD and least often against SpyCEP. In the current study, 60% of the participants in groups 1 and 3 demonstrated antibody increases against either SLO or DNaseB, which is similar to the 67% observed in the previous study. While there is not a standardized ELISA for new immune responses to SLO and DNaseB, these results confirm previous observations showing the relatively low sensitivity of these antibody responses in detecting prior Strep A infection (8, 15, 23). This study also highlights the usefulness of employing a change in the antibody response from one time point to the next for accurately describing a positive association with a particular antigen. This is as opposed to comparison of a single reading against that of ULN values in a population, which may produce false negatives and false positives, given that a rise in titer, although significant, may remain below the ULN and vice versa (Fig. 1).

A recent review proposes the identification of antigenic targets and the development of assays that act as correlates of protection to inform Strep A vaccine development (24). Given the added support from our finding, such an assay would indeed be useful in most settings, especially in remote areas where the burden of disease is highest and overall medical infrastructure is lacking. A major goal of this study was to provide details of the human immune response to five of the Strep A shared antigens (SpyCEP, SLO, SpyAD, SCPA, SSE) that, among others, are currently being evaluated as vaccine candidates (7). As stated in Table 1, the antigens were selected because of their promise as vaccine components and their roles as determinants of Strep A virulence. Protective efficacy of one or more of the shared antigens or M antigens will most likely be determined during vaccine studies in open populations or Strep A challenge studies (25).

Identifying clinical event groups using throat culture and immune response results permitted a more detailed evaluation of the entire data set. There were 28 group 2 events identified in 27 participants, which were defined by a positive throat culture but no new immune response to any of the antigens tested. These participants were more likely to have preexisting high levels of antibodies against shared antigens. Levels of preexisting antibodies in group 2 against SSE, SpyAD, SCPA, SLO, and SpyCEP were notably higher than those in event groups 1 and 3 (Fig. 2). One explanation of these results is that preexisting immunity to one or more of these antigens conferred some resistance to infection but did not prevent acquisition of Strep A in the pharynx. Additional studies will be required to assess mucosal immune responses to multiple Strep A antigens and their role in blocking mucosal acquisition. Alternatively, it is also possible that these individuals experienced a recent infection and continued to carry the Strep A that was recovered from the throat. In support of this is the fact that 22/28 group 2 events were initiated during the enrollment visit of the study, which precluded previous culture or serologic results. However, it is notable that none of the participants demonstrated any increases in antibody levels to any antigens, and only 3 participants had elevated levels of antibodies against the M peptide that corresponded with the *emm* type of Strep A recovered from the pharynx. Based on results from our previous longitudinal study (8), one would predict that >60% of participants with recent infection would mount an antibody response to the homologous M peptide and that the antibody levels would be sustained for some time. Although these results are speculative, they provide important information regarding the human immune responses to shared Strep A antigens that may provide the framework for future clinical vaccine studies.

### Strengths and limitations.

The longitudinal study design provided an opportunity to assess the immune response in sequential samples, mitigating the issues that arise when relying on the upper limit normal (ULN) to describe an increase or decrease to a particular antigen or M-peptide as described by Johnson (15) and Hysmith (8). We also documented a comprehensive profile for all participants throughout their duration in the study, including the acquisition of Strep A symptoms and administration of antibiotics. All samples were processed according to standard protocols, and ELISAs were conducted in two separate laboratories (UCT and UTHSC), to increase the confidence in the results. To the best of our knowledge, this study is the most comprehensive longitudinal analysis of immune responses to Strep A-specific antigens.

There were several limitations regarding the overall study that could potentially affect the conclusions derived from the results. While the sequential sampling provided important insight into the immunological patterns to Strep A antigens for many of the participants, not all demonstrated immune responses are thought to be consistent with a new Strep A infection. For most of the participants included in the ELISA subset of the study, the serologic status prior to the positive Strep A culture at enrollment was unknown. Thus, for those with a Strep A-positive culture on enrollment and elevated levels of M antibodies, it is not possible to determine if these were due to an earlier or the current infection with concomitant increases in antibody levels against one, or more, shared antigens. Second, the two-monthly follow-up period may have been too long between visitations, as the data suggest that there may have been missed opportunities to detect an immune response and/or recover Strep A isolates from the throat. Additionally, there may well have been M peptide responses to alternative *emm* types, as the panel of M peptides selected for this study was based on the recovered isolates only. Lastly, due to the COVID-19 pandemic, we were not able to recruit our original target of 300 participants and, in turn, reduced the number of visits per participant. Moreover, only a subset of the participants was subjected to ELISAs; these were selected based on the acquisition of Strep A, number of visits, and the adherence to the two-monthly follow-up interval. Thus, the completion of more samples in the future may shed some additional insight into the hosts’ immune responses following natural infection with Strep A.

### Conclusions.

This longitudinal study provided insight into the human immune response following pharyngeal Strep A acquisitions in children. Some participants with throat cultures positive for Strep A but no new immune responses to any Strep A antigens were shown to have preexisting high levels of antibodies against some shared antigens. We describe the complex nature of Strep A-specific serum immune responses, including the observation of immunological responses to Strep A antigens in the absence of symptomatic Strep A infections. This may partly explain the disproportionately high burden of acute rheumatic fever and rheumatic heart disease in low-income populations where Strep A acquisition is common but not amenable to antibiotic prevention. Lastly, although the scope of this work was insufficient to confirm the potential of putative vaccine candidates, all antigens tested were in fact immunogenic. This is supportive of future vaccine clinical studies where true human immune correlates of protection may be determined.

## MATERIALS AND METHODS

### Setting and participants.

This longitudinal study was designed to determine the epidemiology and serum immune responses of children (aged 5 to 17) recruited from two juxtaposed peri-urban townships in Cape Town, South Africa. The communities are of lower socioeconomic status and comprise various racial and class groupings. A previous study in South Africa reported children as a major reservoir for Strep A pharyngitis, with an annual incidence of 0.837 cases/100 person-years (unpublished data) and prevalence of approximately 21% in children 5 to 15 years of age (26) in children presenting with sore throat at local clinics.

### Recruitment and enrollment.

The participants were recruited from a local clinic within each township. Individuals in the waiting area of the clinic who presented with a complaint of a sore throat were invited to participate. After informed consent had been obtained from the parent/legal guardian and assent from children >7 years, a questionnaire was completed by the study nurse to capture the demographics. A physical examination was performed, after which a throat culture and blood sample were obtained. Physical findings and symptoms were recorded on a case report form and documented in the AFROStrep RedCap database. Treatment decisions were made by the clinician according to standard practice without input from the research team. The study protocol and procedures were approved by the Ethics Committee of the University of Cape Town and the Institutional Review Board of the University of Tennessee. While this study did not include participants without pharyngitis, a ULN value was determined from sera obtained from “healthy” participants during scheduled follow-up visits, defined as laboratory-confirmed Strep A-negative and asymptomatic for at least 100 days from a previous symptomatic clinic visit.

### Processing of samples.

Throat culture specimens and blood samples were obtained upon enrollment, and subsequent throat swabs and blood samples were requested at two-monthly intervals for a period of 24 months. If a child experienced a sore throat between scheduled visits, they were instructed to return to the clinic for an additional throat culture and serum sample. Throat swabs were sent to the NHLS laboratories for culture to identify β-hemolytic streptococci and for standard serogrouping. Pure cultures were sent to the AFROStrep laboratory for *emm*-typing. Cultures were stored at −80°C until the *emm*-typing procedure.

### Definitions.

Strep A acquisition was described as a throat culture positive for Strep A during the initial enrollment visit. A new Strep A acquisition was defined as a Strep A-positive culture isolated from a participant following the enrollment visit. If Strep A was present in two sequential visits, a new Strep A acquisition was defined if the *emm* types were different. The participants were categorized into three main clinical event groups based on the acquisition of Strep A, immune responses to shared antigens and M peptides, as well as symptoms at the time of infection. He upper limit of normal optical density (OD) values was defined as the 80th percentile of healthy participants.

### *emm*-typing.

The *emm*-typing procedure was performed according to previously published protocols (27 to 29), using three alternative primer sets (27, 30, 31). PCR primers were synthesized at the Department of Molecular and Cell Biology, University of Cape Town, South Africa. The PCR products were sent to the University of Stellenbosch Central DNA Sequencing Facility for sequencing. The DNA sequences generated were submitted to the CDC *Streptococcus pyogenes* database (32) for comparison with existing sequences. The sequence was assigned an *emm* type (e.g., *emm*2) and when necessary, a subtype (*emm*2.1).

### Strep A antigens.

Ten shared Strep A antigens and 25 synthetic M peptide antigens (Peptide2Go; Manassas, VA, USA) were used to assess the human immune responses (Table 1). The M peptides were selected based on the *emm* types recovered from throat cultures in this study. With the exception of DNaseB, the shared antigens were selected based on prior studies indicating that they were potential vaccine antigens. Recombinant proteins were cloned, expressed, and purified as previously described (8).

### Antibody assays.

ELISA was performed as previously described (8). Briefly, all sera were tested in duplicate against each antigen at a concentration of 5 μg/mL. The optimal dilution of test sera was selected for each antigen to yield an OD within the straight-line portion of the ELISA curve, which was determined from preliminary experiments with each antigen using serial dilutions of serum ranging from 1:100 through 1:25,600. The sera were diluted 1:200 for assays with the M peptides, SpyAD, SSE, and Mrp’s. For SLO, SCPA, and DNaseB, the sera were diluted 1:12,800, 1:3,200 and 1:3,200, respectively (8). These dilutions were optimal for detecting the longitudinal change in antibody responses. As a negative control, sera from participants in a previous study (8) were pooled to yield an OD below or equal to the background levels without primary antibody. IVIG was used as a positive control. Positive-control sera against SSE (1/200 dilution) and SCPA (1/3,200 dilution) were included in each assay to assess day-to-day variation of the ELISA. The assays were completed in duplicate in different laboratories to increase the confidence of the data.

The mean OD values from repeated assays were calculated and used as the final OD. All assays were repeated, and results that differed by more than 10% were also repeated. A significant immune response or increase in response was defined by plotting OD values on a standard ELISA curve. As previously determined by Hysmith (8) and Johnson (15), for an OD reading higher than 0.25, a 40% increase in antibody level from the previous visit was considered a significant immune response to that antigen. Preexisting high antibody levels were defined as an OD reading above 0.8 and remained elevated for one or more subsequent visits.
